# Jak3 Is Involved in Dendritic Cell Maturation and CCR7-Dependent Migration

**DOI:** 10.1371/journal.pone.0007066

**Published:** 2009-09-17

**Authors:** Ana Rivas-Caicedo, Gloria Soldevila, Teresa I. Fortoul, Andrés Castell-Rodríguez, Leopoldo Flores-Romo, Eduardo A. García-Zepeda

**Affiliations:** 1 Departamento de Inmunología, Instituto de Investigaciones Biomédicas, México, D.F., Mexico; 2 Departamento de Biología Celular y Tisular, Facultad de Medicina, Universidad Nacional Autónoma de México, México, D.F., Mexico; 3 Departamento de Biología Celular, CINVESTAV, México, D.F., Mexico; Fundação Oswaldo Cruz, Brazil

## Abstract

**Background:**

CCR7-mediated signalling is important for dendritic cell maturation and homing to the lymph nodes. We have previously demonstrated that Jak3 participates in the signalling pathway of CCR7 in T lymphocytes.

**Methodology and Principal Findings:**

Here, we used Jak3^−/−^ mice to analyze the role of Jak3 in CCR7-mediated dendritic cells migration and function. First, we found no differences in the generation of DCs from Jak3^−/−^ bone marrow progenitors, when compared to wild type cells. However, phenotypic analysis of the bone marrow derived DCs obtained from Jak3^−/−^ mice showed reduced expression of co-stimulatory molecules compared to wild type (Jak3^+/+^). In addition, when we analyzed the migration of Jak3^−/−^ and Jak3^+/+^ mature DCs in response to CCL19 and CCL21 chemokines, we found that the absence of Jak3 results in impaired chemotactic responses both *in vitro* and *in vivo*. Moreover, lymphocyte proliferation and contact hypersensitivity experiments showed that DC-mediated T lymphocyte activation is reduced in the absence of Jak3.

**Conclusion/Significance:**

Altogether, our data provide strong evidence that Jak3 is important for DC maturation, migration and function, through a CCR7-mediated signalling pathway.

## Introduction

Dendritic cells are professional antigen presenting cells with a unique ability to activate naive T cells Bone marrow Dendritic cells (BMDCs) are found as precursors in the blood, from where they traffic to different tissues and capture antigen very efficiently [1]. These immature dendritic cells (iDCs) do not exhibit considerable APC's activity; only after antigen contact, the maturation process takes place. These iDC recognize microbial structures such as Pathogen-Associated Molecular Patterns (PAMPs) and Damage-Associated Molecular Patterns (DAMP) using Pattern Recognition Receptors (PRRs). During this process, an increase of MHC-II molecules as well as co-stimulatory molecules, such as CD80, CD86 and CD40, allows DCs to become potent APCs. Additionally, a change in their chemokine receptor expression is important for migration from peripheral tissues, via afferent lymphatics, to the T cell areas of regional lymph nodes (LN) [2], [3].

Chemokines are small chemotactic cytokines (8–10 kDa) that regulate migration and localization of leukocytes in different tissues. Different cell types, including stromal, epithelial and endothelial cells, secrete chemokines, both under inflammatory and homeostatic conditions. Chemokines bind to 7-transmembrane domain G-protein coupled receptors (7TM-GPCR). Among these receptors, CCR7 is highly expressed on mature DCs, and its ligands, CCL21 and CCL19 are expressed in LN and spleen. Moreover, CCL21 is expressed in high endothelium venules (HEV) and in the lymphatic endothelium of multiple tissues[4]. In this context, DCs from CCR7 deficient mice fail to form dermal “cords” at the dermal lymphatic vessels, and show impaired migration into LN. Consequently, these incorrectly localized DCs are incapable to promote appropriate immune responses [5]. Furthermore, CCL19 and CCL21 have shown to be potent stimulators of DC maturation [6]. Additionally, CCL19 optimizes the DC-T cell interaction and increases the frequency of T cell responses to rare cognate antigen [7]. In this context, DCs from mice deficient in both CCL19 and CCL21 chemokines (plt/plt), fail to accumulate in the T cell zones [8] and display defects in DC maturation, as it was demonstrated by a reduction in the expression of costimulatory molecules [6].

Our laboratory and others have demonstrated that the Jak/Stat pathway plays a role in chemokine-mediated signalling [9], [10], [11], [12]. In particular, we have demonstrated that Jak3 is involved in signalling through CCR9 and CXCR4 in murine BM cells and thymocytes [13]. Furthermore, in the absence of Jak3 mature T lymphocytes have impaired CCL19/CCL21-mediated homing to peripheral lymph nodes, mainly due to deficient CCR7 mediated signalling [14].

In this report, we further evaluated the role of Jak3 in DC maturation and migration using a Jak3 deficient (Jak3^−/−^) mouse model. We found that BMDCs derived from these mice show impaired migration towards CCL19 and CCL21. In addition, *in vitro* maturation of BMDCs induced by LPS was significantly affected and Jak3^−/−^ BMDCs showed reduced ability to stimulate allogeneic T cell proliferation responses. Moreover, contact hypersensitivity assays (CHS) performed in Jak3^−/−^ mice showed a significant reduction in ear thickness, compared to wild type mice, which correlated with decreased Langerhans cells (LC) recruitment at the induction zone after challenge and a reduced inflammatory response. Our data suggests that Jak3 may play an important role in regulating DC functions *in vivo*, both in homeostasis and under inflammatory conditions.

## Results

### Dendritic cell maturation is impaired in the absence of Jak 3

**Figure 1:**
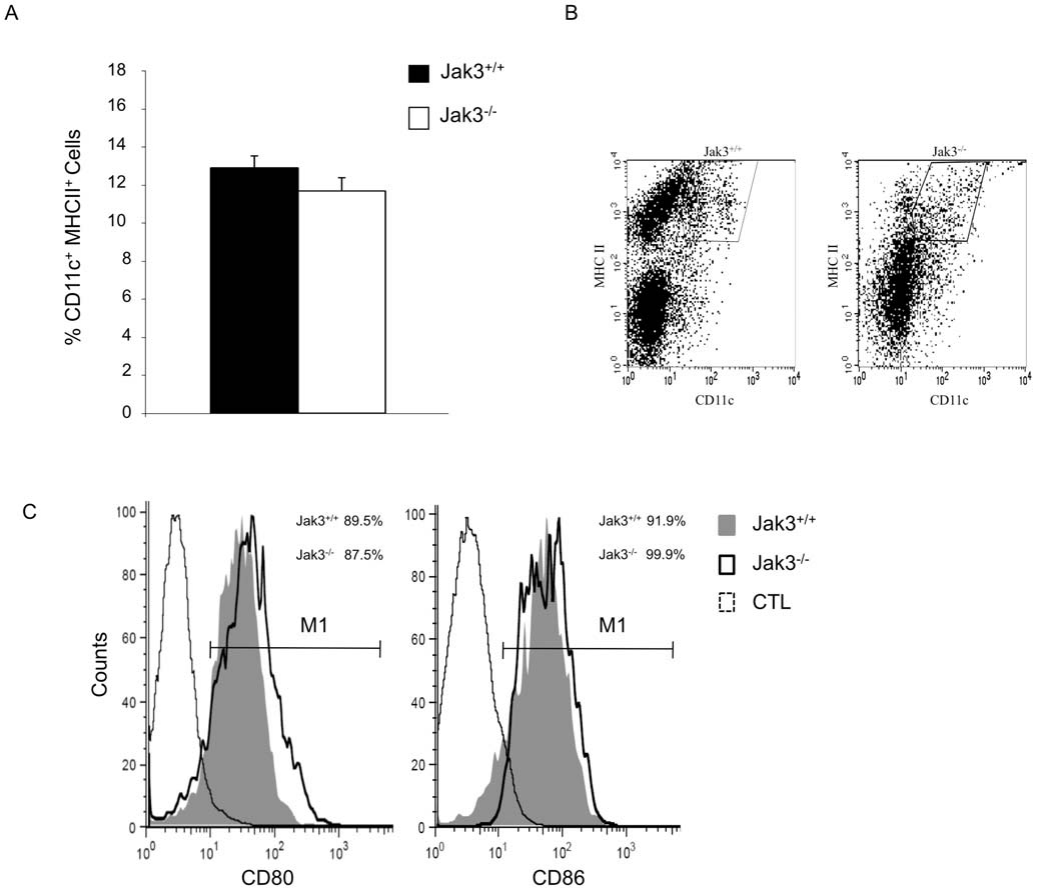
Normal Dendritic cell development in Jak3^−/−^ mice. FACS analysis from cells isolated from Jak3^+/+^ and Jak3^−/−^ spleen were stained with anti-CD11c, anti-MHCII, anti-CD80 and anti-CD86 antibodies. A) Histogram representing the percentage of cells expressing CD11c^+^ MHCII^+^. Data represent mean values ±SEM (n = 9). B) A representative dot plot analysis of the expression of CD11c^+^ MHCII^+^ in DC cells. C) Analysis of maturation markers CD80 and CD86. A representative experiment is shown. CTL: isotype control. M1 indicates the region used to calculate the % of positive cells for each costimulatory molecule.

To test whether the absence of Jak3 affects the *in vivo* generation of DCs, phenotypical FACS analysis of cells isolated from Jak3^−/−^ spleen was performed using anti-CD11c, anti-MHC Class II, and anti-CD80 or anti-CD86 antibodies. As shown in Figure 1A, no differences were observed in the percentage of DCs present in Jak3^−/−^ compared to Jak3^+/+^ mice. In addition, analysis of the maturation markers showed that DCs from Jak3^−/−^ mice expressed higher levels of CD80 (p = 0.047), **consistent with a previous report** [**15**], although CD86 levels were not significantly different in the Jak3^−/−^ mice compared to Jak3^+/+^ mice (p = 0.45) (Fig. 1B, **and supplementary Figure S1**).

**Figure 2:**
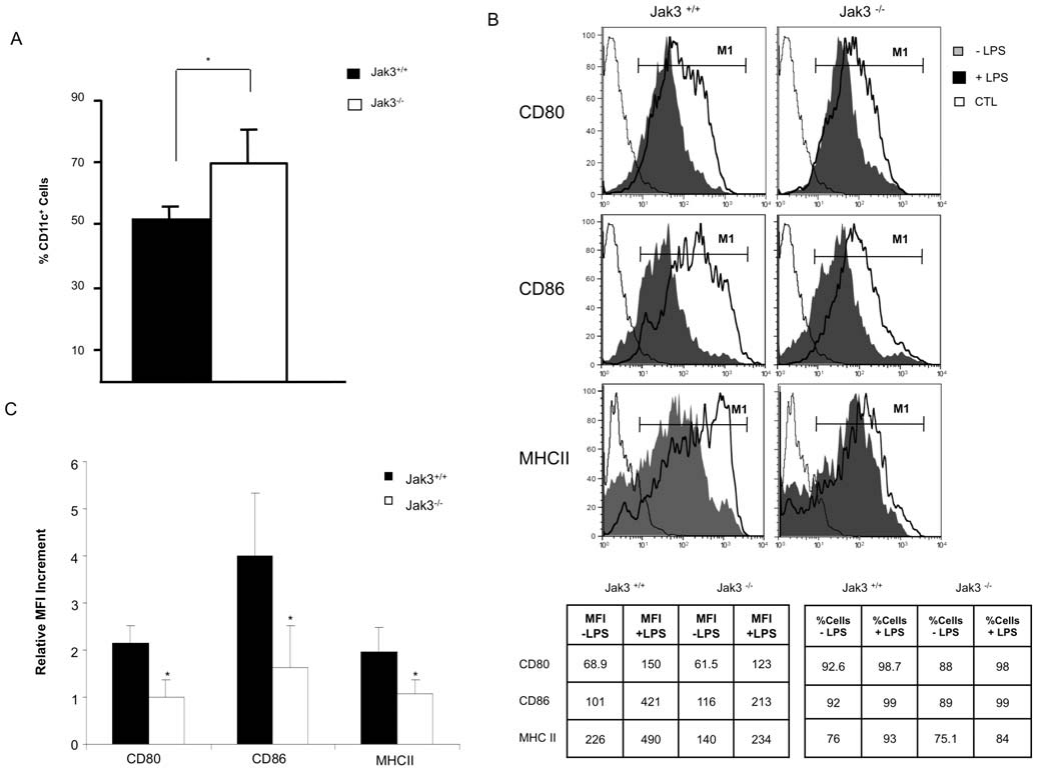
Impaired maturation of Jak3^−/−^ Bone Marrow derived Dendritic Cells. DCs were derived from Jak3^+/+^ and Jak3^−/−^ bone marrow progenitors and stimulated with LPS (1 µg/ml) to obtain mature BMDCs (mBMDCs) as described in Methods. mBMDCs were stained using anti-CD11c, anti-MHC-II, anti-CD80 and anti-CD86 antibodies. A) Histograms representing % of CD11c cells in BMDCs from Jak3^+/+^ (filled) and Jak3^−/−^ (empty) (*p≤0.05). B) Analysis of the expression of costimulatory molecules in BMDCs from Jak3^+/+^ and Jak3^−/−^. Filled histogram represent mBMDCs, grey histogram represent iBMDCs and empty histogram, control antibodies. On the right panel, the Table summarizes the data obtained in this representative experiment, indicating the % of CD11c^+^ and the MFI values of iBMDC and mBMDC from Jak 3^+/+^ and Jak 3^−/−^ mice, respectively. C) Mean fluorescence intensity increment of the expression of maturation markers after LPS stimulation. This value was calculated as the increment in MFI from mBMDCs, relative to the value from iBMDCs as described [41]. Results are shown from a representative experiment (n = 14; *p≤0.05).

Next, to analyze the capacity of Jak3^−/−^ mice to generate DCs *in vitro*, BM cells were obtained and cultured in the presence of GMCSF, as described in materials and methods, LPS was used to induce DC maturation and expression of CD80, CD86 and MHC Class II was analysed by FACS. Analysis of immature BMDCs (iBMDCs) showed that Jak3^−/−^ BM cells were capable to induce the differentiation of CD11c^+^ cells *in vitro* to the same extent as Jak3^+/+^ cells (data not shown). Unexpectedly, after LPS stimulation, the percentage of CD11c^+^ cells in culture was significantly higher in BMDCs derived from Jak3^−/−^ mice, compared to wild type mice (Fig. 2A). However, FACS analysis showed that after stimulation with LPS, Jak3^−/−^ BMDCs had impaired up-regulation of maturation markers, MHC Class II, CD80 and CD86 (Fig. 2B and Fig. 2C). These results suggested that in the absence of Jak3, their maturation process might be compromised.

### Impaired chemotaxis of Jak3^−/−^ DCs towards CCL19 and CCL21 chemokines

**Figure 3:**
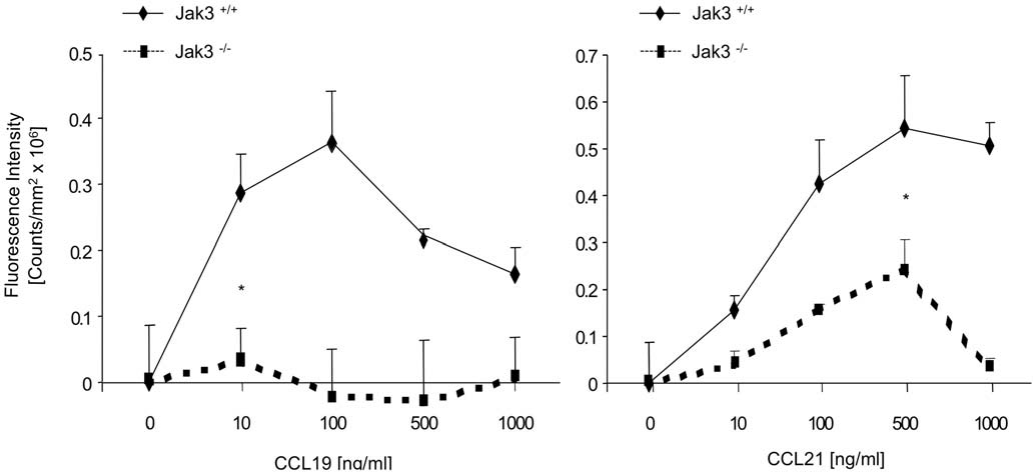
Analysis of chemotaxis of Jak3^−/−^ DCs towards CCL19 and CCL21 ligands. An *in vitro* chemotaxis assay was performed using mBMDCs (24 h after LPS stimulation) from Jak3^+/+^ and Jak3^−/−^ mice. 2×10^6^ cells/ml were resuspended in the chemotaxis buffer with Ca^++^ and Mg^++^, and allowed to migrate towards different concentrations of CCL19 and 21 chemokines. Analysis of migrating cells was performed as described in methods. In the Y-axis is represented the fluorescence intensity (total counts) corresponding to migrating cells. This figure shows one representative of four independent experiments (n = 12; *p≤0.05).

**Figure 4:**
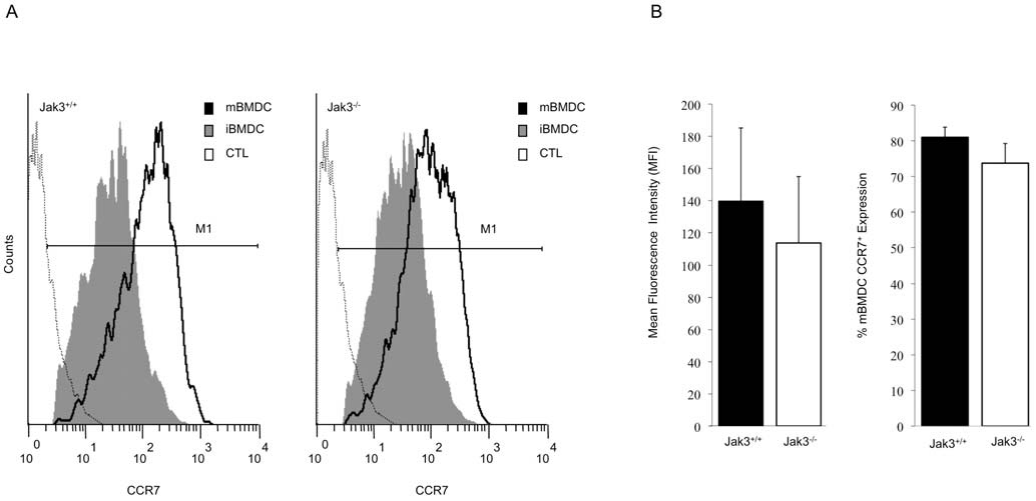
Jak3^−/−^ DCs express normal levels of CCR7 on their surface. Immature and mature BMDCs from Jak3^+/+^ and Jak3^−/−^ mice were labelled with a CCL19-Fc fusion protein followed by anti-hIgG-FITC and analyzed by flow cytometry to determine CCR7 levels on the CD11c^+^gated subpopulations. A) Analysis of expression of CCR7 in iBMDC and mBMDCs from Jak3^+/+^ (thick line) and Jak3^−/−^ (grey shadow). Thin line represents the secondary antibody control (CTL). B) Analysis of the iBMDC and mBMDC mean fluorescence of CCR7^+^ and % of mBMDC CCR7^+^ cells of Jak3^+/+^ and Jak3^−/−^ mice. Data represent the mean of three different experiments (n = 6; *p<0.05).

CCR7 is important for DCs migration both under inflammatory and homeostatic conditions [5]. Both CCR7 ligands, CCL19 and CCL21, are expressed in lymphatic organs, although only CCL21 is expressed in lymphatic vessels. Arrival of DCs to LNs is guided by the interaction with these chemokines. Earlier, we have reported that Jak3 was involved in CCR7-mediated T lymphocyte homing to peripheral LNs [14]. To further study the role of Jak3 in CCR7-dependent DC migration, we analyzed the migration capacity of mature BMDCs (mBMDCs) derived from Jak3^−/− and^ Jak3^+/+^ mice using *in vitro* chemotaxis assays. BMDCs from Jak3^+/+^ showed efficient migration to CCL19 and CCL21 at concentrations ranging between 10 and 500 ng/ml, before (iBMDCs, data not shown) and after (mBMDCs) addition of LPS. In contrast, mBMDCs from Jak3^−/−^ mice showed negligible chemotactic responses to both chemokines (Fig. 3). To exclude the possibility that impaired migration was due to deficiencies in CCR7 expression on Jak3^−/−^ cells, we analysed its expression by FACS. No significant differences were found between Jak3^+/+^ and Jak3^−/−^ BMDCs; approximately 80% of CD11c^+^ cells were CCR7^+^ after LPS stimulation in both Jak3^+/+^ and Jak3^−/−^ mice (Fig. 4A). The mean fluorescence intensity of CCR7^+^ cells was not significantly different in either iBMDC (not shown) or mBMDC from Jak3^+/+^ and Jak3^−/−^ mice (Fig. 4B). This result indicated that impaired DC migration was rather **due** to CCR7 deficient signalling than expression in the absence of Jak3.

### Jak3 is not necessary for the migration of skin DCs (Langerhans cells) from epidermis to dermis, but is required for seeding regional lymph nodes

**Figure 5:**
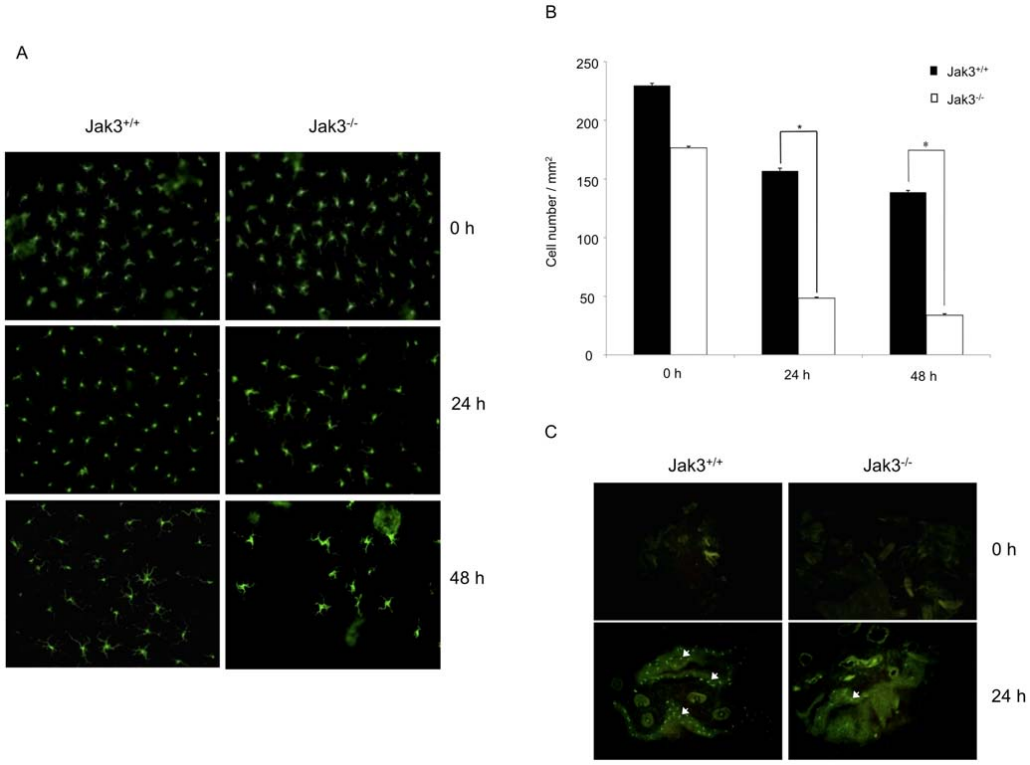
Normal LC migration from the epidermis but abnormal localization around dermal lymphatics in Jak3^−/−^ mice. A) Ears from Jak3^+/+^ and Jak3^−/−^ mice were separated into dorso-ventral halves and the whole skin was cultured in RPMI 10% FBS. At different times, pieces were removed and the dermis was separated from the epidermis to get epidermal sheets, as described in Materials and Methods. They were fixed in acetone and stained with anti-Langerin followed of an anti rat IgG-FITC antibody. Figure represents 40× magnification. B) The number of LC in the skin after the culture was quantified. Data represent mean values ± SEM (n = 6; *p≤0.05). C) Whole cultured skin was frozen and cryostat sections were obtained to expose the epidermis and dermis. Sections were stained overnight with anti-Langerin followed by anti-rat IgG FITC. DCs in the Jak3^+/+^ mice are located around lymphatic vessels forming dermal cords; these are not visible in Jak3^−/−^ mice.

Langerhans cells (LC) are a type of DCs present in the skin, whose function is to capture antigen and migrate to lymph nodes, via afferent lymphatics, where they stimulate naive T cells. The first step for LC mobilization is their migration from epidermis to dermis; this process is not dependent on CCR7, as it was demonstrated by means of skin explants cultures [5]. Similarly, we analyzed the role of Jak3 in regulating LC migration using a mouse ear skin model. In this system, ear skin was floated directly on culture medium (dermis down). After 24 h and 48 h, epidermis and dermis were separated, to identify LCs, epidermis was stained with an anti-Langerin antibody. As shown in Fig. 5A, reduced numbers of LCs were present in the skin from Jak3^−/−^ mice after 24–48 h of culture, compared to Jak3^+/+^ mice. To exclude the possibility that this reduction was the result of a decrease in Langerin expression, we also analysed the epidermis by using anti-MHC Class II staining (data not shown). These observations may indicate that, in the absence of Jak3, LCs exit the epidermal compartment at a faster rate compared with Jak3^+/+^ mice (Fig. 5B). Once DCs enter the dermis, they need to adhere, likely through lymphatic formations known as dermal cords. We analysed this process and investigated whether in the absence of Jak3, chemokine receptor-mediated signalling had an effect on the formation of these cords. Accordingly, after 48 h culture, the presence of structures resembling dermal cords was evident in skin explants from Jak3^+/+^ mice. On the contrary, Jak3^−/−^ explants showed no evidence of these structures, similarly to that reported in the CCR7 mice^−/−^ (Fig. 5C).

**Figure 6:**
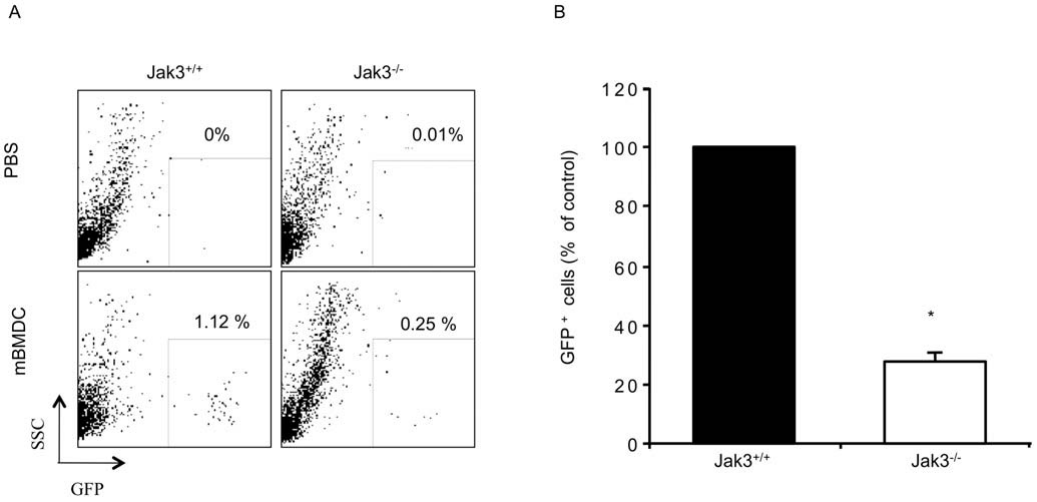
Jak3^−/−^ DCs showed impaired homing to the lymph nodes after adoptive transfer. Mature BMDCs were obtained from Jak3^+/+^-GFP and Jak3^−/−^-GFP mice and injected into the footpad of Jak3^+/+^ mice, as described in Methods. At different time points, popliteal LNs were collected and the percent of GFP^+^ cells were determined by FACS analysis. A) Representative dot plot of three individual experiments at 36 h after transfer. B) Data represent the mean of three independent experiments 36 h after transfer (n = 9, *p<0.05).

To further verify that Jak3 deficiency may also affect *in vivo* migration of DCs, Jak3^−/−^ mice were crossed to Green Fluorescent Protein (GFP)-Jak3^+/+^ mice to obtain GFP^+^-Jak3^−/−^ mice. BMDCs were derived from both GFP^+^-Jak3^+/+^ and GFP^+^-Jak3^−/−^ mice and allowed to maturate *in vitro*, as before. These mBMDCs were injected into the right footpad of Jak3^+/+^ mice. The left footpad was injected with PBS and used as control. Popliteal LN were obtained at different times and the percentages of GFP^+^ cells were analyzed by flow cytometry. GFP^+^ Jak3^+/+^ mBMDCs were identified in the draining LN 24 h after transfer, with a peak between 36 h and 48 h. In contrast, the number of GFP^+^ -Jak3^−/−^ DCs emigrants was reduced, **compared to Jak 3^+/+^ DCs** (Fig. 6A and 6B), remaining low even at 72 h after the transfer (data not shown). This evidence demonstrates that Jak3 has a role in regulating migration of mBMDCs to the LN. In this context, it has been reported that DCs from CCR7^−/−^ mice fail to migrate into the lymph nodes [3], [5]. This data supports the hypothesis that Jak3 is necessary for both *in vitro* and in *vivo* CCR7-dependent migration of DCs.

### Reduced proliferative T cell responses induced by Jak3^−/−^ DCs

**Figure 7:**
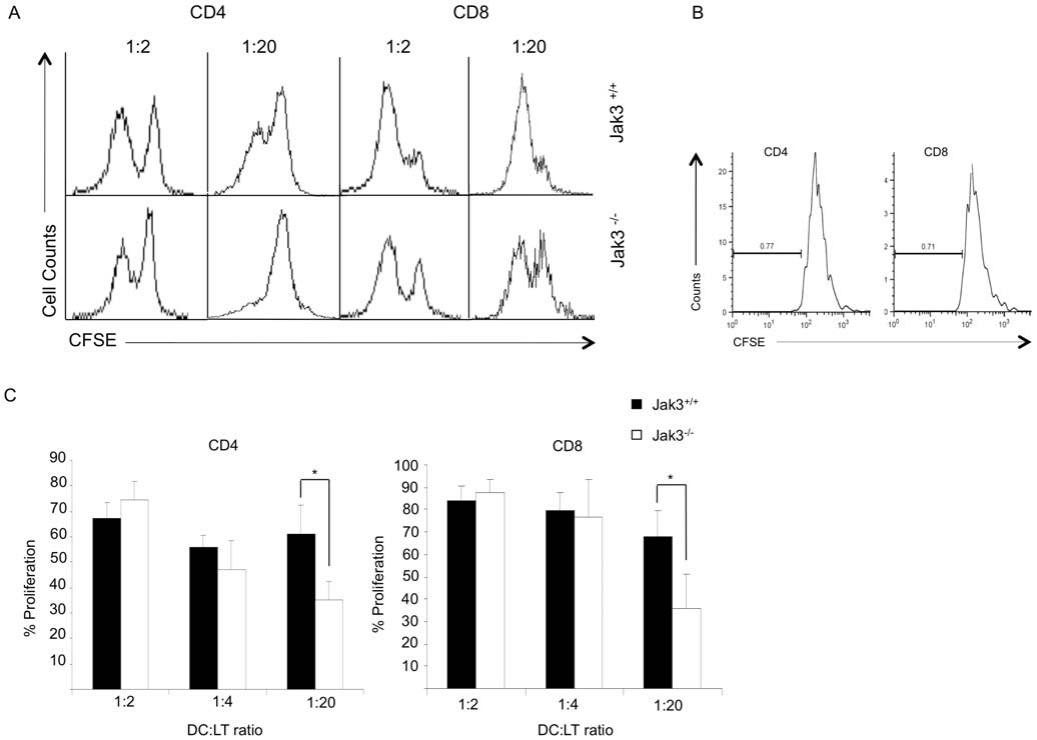
Reduced T lymphocyte proliferation responses induced by Jak3^−/−^ DCs. 1×10^5^ T lymphocytes from Jak3^+/+^ Balb/c AnN mice were CFSE-labelled and co-cultured with Jak3 ^+/+^ or Jak3 ^−/−^ mBMDCs in RPMI 10% SFB for 5–7 days at different DCs: T cell ratios (1∶2, 1∶20), as described in Methods. Cells were harvested from the cultures and stained with anti-CD4 and anti-CD8 antibodies. A) A representative experiment is shown. B) Negative control for T cell proliferation, in unstimulated CD4^+^ or CD8^+^ gated subpopulations. C) Data represents the percentage of proliferation at different cell ratios expressed as mean values ± SEM (n = 6; p≤0.05).

One of the hallmarks of DC's functions is T lymphocyte activation. Since Jak3^−/−^ mBMDC showed reduced expression of maturation markers after LPS stimulation, it was of interest to know whether this function was affected. Using a MLR assay, T lymphocytes were stimulated with DCs from both Jak3^+/+^ and Jak3^−/−^ mice at different ratios. Our assays showed that DCs from Jak3^−/−^ mice were able to stimulate similar proliferative responses of T lymphocytes at ratio of 1∶1 to 1∶4 (DC: TL). In contrast, at a ratio of 1∶20, BMDCs from Jak3^−/−^ mice promoted a weak proliferation of CD4 and CD8 T lymphocytes compared to Jak3^+/+^ (Figs. 7A and 7C).

### Analysis of in vivo migration of DCs by a Contact Hypersensitivity (CHS) assay

**Figure 8:**
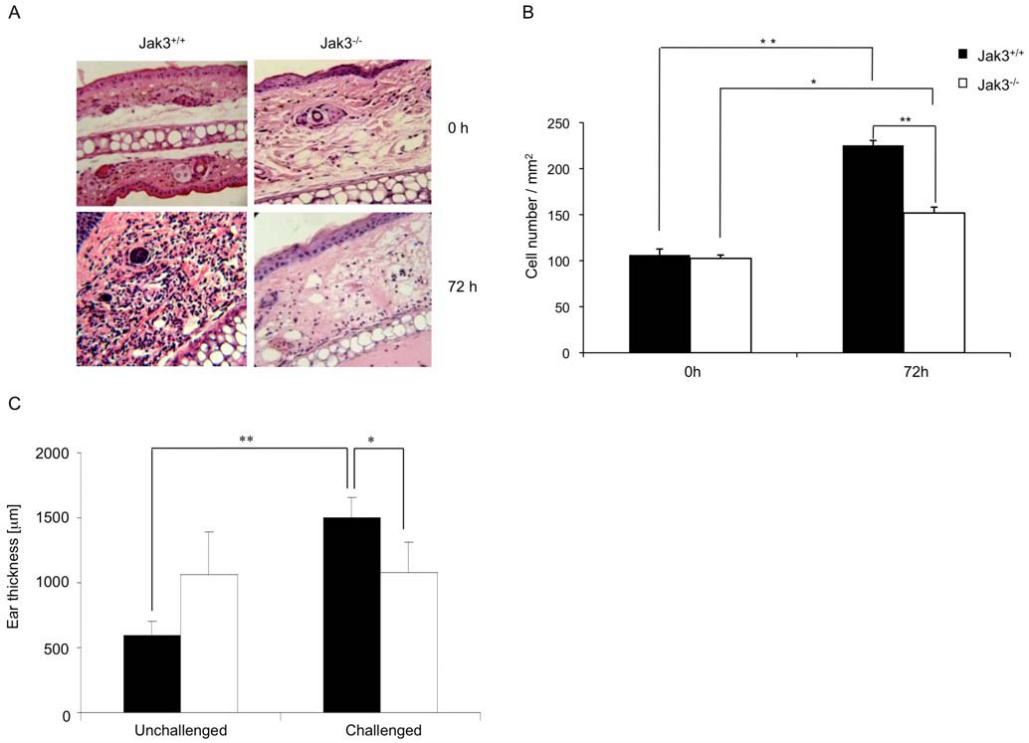
Jak3^−/−^ mice show reduced CHS responses. Mice were sensitized in the right ear with 10 µl of 1% DNFB-olive oil and five days after challenged with the same hapten solution. Frozen sections were obtained at different time points and a Haematoxylin/Eosin staining was performed. A) H&E staining of ear skin at 0 and 72 h after challenge from a representative experiment is shown. B) Total cell numbers per mm^2^ were quantified in the dermis after challenge. C) The ear thickness from the perichondrium to basal membrane after the challenge was calculated. Data represents the mean of four independent experiments ± SEM (n = 3). Magnification 40× (*p≤0.05; **p≤0.01).

**Figure 9:**
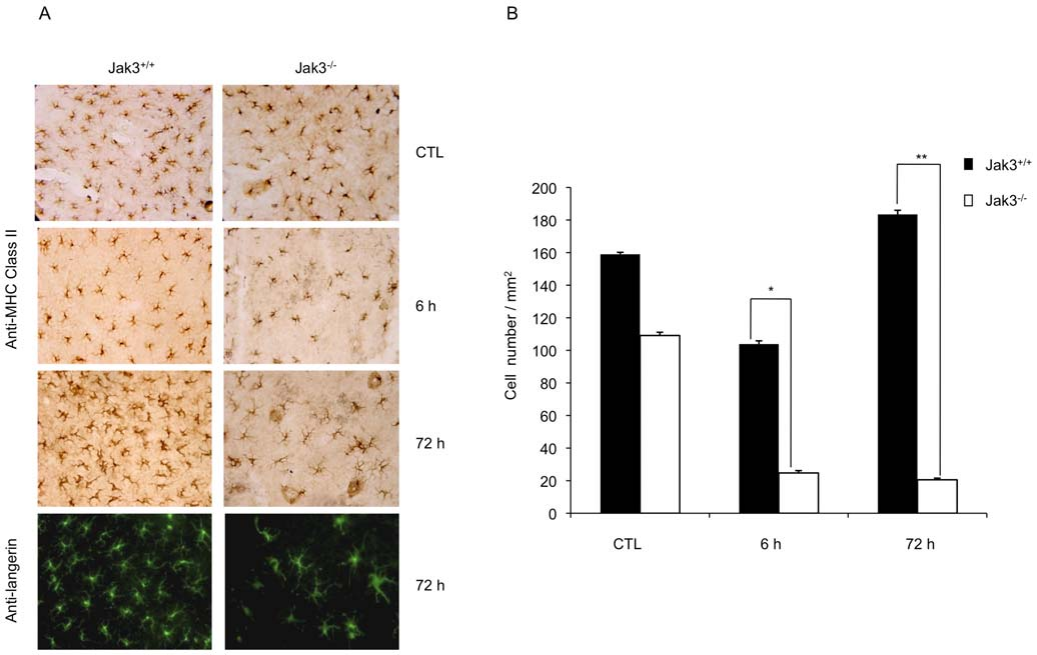
Jak3 has a role in the restoration of LC population at the epidermis. After challenge, epidermis and dermis were separated at different time points as described in materials and methods. A) The epidermis was stained at 6 and 72 h after challenge using an anti-MHCII antibody followed by streptavidin-HRP (upper panels). Staining with anti-Langerin and anti-FITC labelled secondary antibody was also performed (lower panel). B) Cell number was quantified and data represent the mean of three individual assays performed in triplicate (*p≤0.05; ** p≤0.01).

Next, based on the results obtained in the *in vitro* chemotaxis and *in vivo* adoptive transfer assays, we decided to test whether Jak3 was involved in LC migration during a CHS response. A CHS response is a T-cell mediated skin inflammation reaction resulting from exposure to haptens. DNFB was used as hapten to induce CHS in both Jak3^+/+^ and Jak3^−/−^ mice. A 1% DNFB solution was applied onto the left ear of each mouse. Five days later, the right ear was challenged with the same solution. Ear skin was obtained and initially stained by H & E (Fig. 8A); total cell number was quantified at different time points. This analysis demonstrated a statistically significant difference between Jak3^+/+^ and Jak3^−/−^ mice in the number of total cells recruited after 72 h of challenge (Fig. 8B). At 6 and 72 h after challenge, ear thickness in both Jak3^+/+^ and Jak3^−/−^ mice was also measured and compared with ear thickness unchallenged. There was a considerable increase in the size of the treated ear from Jak3^+/+^ compared to Jak3^−/−^ mice at 72 h after challenge (Fig. 8C). **This data demonstrate that the absence of Jak3 results in a defect in cell recruitment under inflammatory conditions.** In fact, *in vivo* adoptive transfer of mBMDCs showed that, in the absence of Jak3, mBMDCs were greatly impaired in their homing to LNs (Fig. 6A). Additionally, to identify LCs, epidermal sheets were stained with anti-Langerin and Anti-MHC Class II antibodies (Fig. 9A). As shown, 6 h after challenge, epidermal sheets from Jak3^+/+^ mice showed a decrease in the number of LC, which was even lower in Jak3^−/−^ mice. However, 72 h after challenge, the percentage of Jak3^+/+^ LCs was already recovered while it was very low in the epidermal sheets from Jak3^−/−^ mice (Fig. 9B), suggesting that Jak3-mediated signalling is part of a complex mechanism regulating migration of LCs **(see scheme in supplementary Figure S2)**.

## Discussion

The Jak/Stat pathway has been shown to be an important regulator of chemokine receptor signalling [16], [17]. Specifically, it was reported that different members of the Janus family of kinases (Jak1, Jak2, Jak3 and Tyk2), become tyrosine phosphorylated upon stimulation with various chemokines in different cell types [reviewed in [18].

Our laboratory has previously shown that Jak3 is involved in CCR9 and CXCR4 receptor signalling in bone marrow progenitors and thymocytes [13] and more recently, that phosphorylation of Jak3 in peripheral lymphocytes in response to CCL19 and CCL21 is important for T lymphocyte homing to peripheral lymph nodes [14], which may partially explain the absence of peripheral lymph nodes and Peyer's patches in Jak3^−/−^ mice.

In this report, we investigated the role of Jak3 in regulating DC migration and function. We found that in the absence of Jak3, mice were capable to generate DCs *in vivo*, since normal numbers of CD11c^+^ MHC Class II ^+^ cells were found in the spleen of these mice. This is in contrast to a previous report showing decreased numbers of splenic DCs in Jak3^−/−^ mice [15]. In addition, when we compared *in vitro-*generated DCs from bone marrow progenitors (iBMDCs) no quantitative differences were observed between Jak3^−/−^ mice and Jak3^+/+^ mice. Interestingly, *in vitro* LPS-induced maturation of BMDCs led to significantly higher numbers CD11c^+^ cells in the absence of Jak3^−/−^, which correlates with the fact that Jak3 has been shown to act as a negative regulator of DC survival [15]. However, we found that DCs maturation after LPS stimulation is strongly affected in Jak3^−/−^ BMDCs, since the increase of surface CD80, CD86 and MHC Class II molecules was clearly impaired in Jak3^−/−^ DCs compared to Jak3^+/+^ cells. This result contrasts with a previous report showing that maturation of DCs is not affected in the absence of Jak3. **However, in this report the authors used Ftl3-L to derive DC from bone marrow, and in addition they used CD40 cross-linking instead of LPS, as maturation stimulus. Finally this report only reported changes in the expression levels of CD86** [**15**] **, while we have also analyzed MHC Class II and CD80 expression.**

There is growing evidence showing that chemokines and chemokine receptors participate in the maturation process of DCs. CXCL12/CXCR4 has been involved in promoting maturation and survival of BMDCs. Their involvement was demonstrated by using CXCR4 antagonists in BMDCs cultures and antigen-specific *in vitro* proliferation assays [19]. In addition, analysis of lung DCs obtained from CCR5^−/−^ mice, demonstrated that they are less efficient capturing FITC-dextran and express fewer levels of CD86 and MHC Class II molecules compared to wild type mice [20]. Another chemokine, CCL16, induces an increase in the expression of CD80 and CD86 molecules on DCs through a process that involves p38 and PLC signalling [21]. More interestingly, CCL19 and CCL21 have recently been involved in DC maturation, by enhancing the up-regulation of CD86 and CD40 expression markers, whereas CCL19 stimulation also led to increased secretion of IL-12, IL-1β and TNFα [6] from DCs. In this context, our group has recently reported that CCL19 and CCL21 induce Jak3 phosphorylation in T lymphocytes [14]. Thus, a mechanism by which the lack of Jak3 could negatively affect their maturation might be through a deficiency in the CCR7 signalling pathway. In contrast, expression of costimulation markers on mature DCs after stimulation with IL-4 appeared to be independent of Jak3. However, the production of IL-12, p70, induced by microbial products was shown to require Jak3 [22]. Altogether, these findings suggest that Jak3 might not be directly involved in the DC development but rather it may regulate their maturation and effector functions.

A requirement for DC function is their ability to migrate to peripheral lymph nodes, where they undertake a key role during the priming of naive T lymphocytes. Migration of mature DCs to LN occurs through the afferent lymphatics and has been shown to involve CCR7-mediated signalling [23], as assessed by the decreased homing of DCs into the lymph nodes observed in CCR7^−/−^ mice [24] or in *plt/plt* mice [8]. Furthermore, CCR7 was shown to be a key regulator that governs trafficking of skin DCs under both inflammatory and steady state conditions [5]. Our group has previously demonstrated that Jak3 is required for CCR7 mediated T lymphocyte homing to peripheral LN [14]. In this study, we analyzed the role of Jak3 in the migration of mature DCs both *in vitro* and *in vivo*. We found impaired migration of Jak3^−/−^ BMDCs towards CCL19 and CCL21. Our *in vitro* experiments showed that Jak3^−/−^ mature DCs have lower migration towards CCL19 and CCL21 compared to Jak3^+/+^ mature DCs. These data indicate that the impaired migration of Jak3^−/−^ DCs must be due to CCR7-deficient signalling in the absence of Jak3. More importantly, *in vivo* adoptive transfer assays using mature GFP^+^-BMDCs, showed that Jak3^−/−^ DCs arrival to LN is reduced in about 80% compared to Jak3^+/+^ DCs. Taken together, these results may imply an important role of Jak3 in the CCR7-mediated signalling during the migration of DCs into the lymphatic vessels.

To further assess the relevance of the Jak3 expression in DCs migration, we developed an ex-vivo model of migration using whole ear skin cultures. It has been described that chemokines and other molecules such as CD47[25], prostaglandins [26], [27] and leukotrienes [28] regulate the exit of LC from skin and their later arrival into the lymph nodes. In the epidermis DCs interact with keratinocytes through E-cadherin homophilic interactions [29]. It is known that molecules such as JAM-A are also involved in retaining DCs at the epidermis [30], whereas ICAM-1 expressed in the lymphatic endothelium is involved in the entry of LC into lymphatic vessels [31]. Interestingly, although it has been reported that Jak3 might be involved in the development of human epidermis [32], Jak3^−/−^ mice showed no **apparent** alterations in the epidermis (Figures 5B and 9B); indeed, these mice displayed normal numbers of LC compared to Jak^+/+^ mice. Furthermore, our results demonstrated that DCs from Jak3^−/−^ mice are fully capable of leaving the epidermis.

DCs leaving the epidermis into the dermis locate along the lymphatic vessels of the dermis to form the so-called “cords”. The formation of these structures may be of relevance during the entry process into these vessels. Interestingly, CCR7^−/−^ DCs fail to form these “cords” within the dermis [5]. Similarly, our data showed that DCs from the Jak3^−/−^ are greatly impaired in their ability to form these dermal “cords”.

In addition, we analysed the ability of DCs to induce T cell proliferation in the absence of Jak3. We found that allogeneic T lymphocyte proliferation is significantly reduced after contact with Jak3^−/−^ DCs when a ratio 1∶20 (DC: T cell) was used compared to the proliferation obtained with Jak3^+/+^ DCs at the same ratio. This decreased proliferation **might be explained by** a defective expression of costimulatory molecules in the Jak3^−/−^ DCs and/or to decreased DCs cytokine production. When increased ratios of Jak3^−/−^ DC: T cell were used, normal levels of proliferation were obtained, indicating that increasing the number of DCs can compensate the difference in the levels of costimulatory molecule expression. Since only 10% of the total T lymphocyte repertoire recognizes MHC alloantigens, increasing the number of MHC-peptide complexes augmented the efficiency of peptide recognition on the allogeneic DCs. In this context, previous reports from others and from our laboratory (data not shown) using an OVA TCR-specific transgenic system, reported no significant differences in T lymphocyte activation in the absence of Jak3 [15].

Next, to evaluate *in vivo* DC-mediated functions, CHS assays using DNFB were performed. A subsequent antigen challenge induces activation of macrophages and lymphocytes primarily, leading to inflammation. To reach the lymph nodes, the DCs should enter the lymphatic vessels. The evidence that Jak3^−/−^ DCs were unable to form “cords” around the lymphatic vessels and that they did not reach the lymph nodes after adoptive transfer, is consistent with the fact that inflammation in the CHS responses, measured as the ear thickness obtained after challenge, was always lower in the skin of Jak3^−/−^ mice compared to that of Jak3^+/+^ mice. Interestingly, even though untreated Jak3^−/−^ mice consistently presented thicker ears than Jak3^+/+^ mice, ear thickness did not increase after challenge in the Jak3^−/−^ mice. In addition, although challenge of Jak3^−/−^ mice with DNFB induced significant cell recruitment at the site of inflammation compared to mice treated with the vehicle, the cell counts were much lower that those seen in Jak3^+/+^ mice. These results correlate with the *in vivo* data showing a decrease in the number of migrating DCs reaching the draining LN, which may result in decreased T cell activation. In addition, since Jak3^−/−^ mice lack peripheral LN, it is likely that cognate T lymphocyte activation takes place in another secondary lymphoid organ, such as the spleen. In this context, in the *plt/plt* mice, where entry of T lymphocytes and DCs to the LN is greatly impaired, a proportion of T cell responses are shifted to the spleen. Interestingly, in these mice responses to contact sensitization were decreased at day 2 after priming but increased at day 6, leading to enhanced T cell responses [33].

We also determined the Langerhans cell numbers at the site of inflammation, after challenge, in the CHS assays. Under steady-state conditions, LCs leave the skin with a low but constant frequency. These cells might be maintained by a stable and renewable precursor population in the skin [5], [34], [35]. Conversely, under inflammatory conditions, this subpopulation is replaced by blood-borne LC progenitors [35], [36]. These precursors are Gr1^+^ monocytes, which are incorporated into the epidermis where they become Langerin positive and acquire the morphology characteristic of LCs [36]. In our assays, we found that after antigen challenge, Jak3^−/−^ LCs did not repopulate the epidermis with the same efficiency that those from Jak3^+/+^ mice, which suggests that this Jak3 kinase might be involved in this process.

Altogether, the deficiencies in maturation and migration induced through CCR7 in Jak3^−/−^ DCs allow us to conclude that Jak3 is involved in both processes; hence its absence affects negatively both the DC migration and **DC-mediated functions**.

## Materials and Methods

### Ethics Statement

All animals were handled in strict accordance with good animal practice as defined by the Animal Experimental Bio-Ethics Guidelines of the Instituto de Investigaciones Biomédicas. All animal work was approved by the Animal Experimental Bio-Ethics Committee of the Instituto de Investigaciones Biomédicas, UNAM.

### Mice

C57BL/6, Jak3-deficient mice (Jak3^–/–^) (C57BL/6-JAK3tm11jb) were obtained from The Jackson Laboratories (Bar Harbour, ME) and Balb/c AnN mice were obtained from the animal facility at the Instituto de Investigaciones Biomédicas (UNAM, Mexico) and kept in pathogen-free conditions. Actin-green fluorescent protein (GFP) transgenic mice on the C57BL/6 background were obtained from Dr M. Okawa (Genome Information Research Center, Osaka University, Japan). These mice were crossed with Jak3^–/–^ mice for a few generations in order to obtain actin-GFP transgenic mice on the Jak3^–/–^ background. Six to ten week-old homozygous Jak3^–/–^mice were used. In some experiments C57BL/6 age-matched mice (wild type) were used as controls.

*In vitro* differentiation of Bone Marrow derived Dendritic Cells. BM derived Dendritic cells were generated and derived as described earlier [37]. Briefly, BM was extracted from the femurs and tibias of Jak3^+/+^ and Jak3^−/−^ mice. Red cells were lysed with a lysis buffer (0.83% ammonium chloride) and 5×10^6^ BM cells were derived after culture in 10% FBS containing RPMI, supplemented with 15% of supernatant from the GMCSF stably transfected CHO cell line [38]. After 5 days, cells were analyzed for expression of Class II and CD11c (iBMDC) by flow cytometry. To induce mature BMDC (mBMDC), cells were stimulated with 1 µg/ml LPS (*E. coli* 0111:B4, Sigma Chemical, St. Louis, MO) for 24 h and phenotypically assessed by flow cytometry, as before. BMDCs were identified by gating the CD11c^+/high^ Class II^+^ subpopulation, as previously described [39].

### Flow Cytometry

Single-cell suspensions from spleen were prepared as previously described [14]. Cells were stained with PE-labelled anti-CD86, PE-labelled anti-CD80 and FITC-labelled anti-CD11c (BioLegend, San Diego, CA), PE-labelled anti-CD11c (Becton Dickinson, San Jose, CA), biotin-labelled anti I-A/I-E (2G9, Becton Dickinson) **followed by streptavidin-cychrome (Becton Dickinson)**. To assess CCR7 expression, a CCL19-Ig fusion protein (generously provided by Dr. U. von Andrian, Harvard University, Boston, MA) was used as previously described [14], followed by a FITC-labelled goat anti-hIgG secondary antibody. **For biotin-conjugated antibodies, we used cells stained with streptavidin–cychrome alone (Becton Dickinson) as a negative control. For directly conjugated monoclonal antibodies, unstained cells were used as controls, and staining with an irrelevant monoclonal conjugated antibody**. Cells were acquired in a FACSCalibur flow cytometer (Becton Dickinson) and data was analyzed using Cell Quest Pro software (Becton Dickinson).

### *In vitro* migration of Bone Marrow derived Dendritic Cells

2×10^6^ BM cells were obtained from Jak3^−/−^ or Jak3^+/+^ mice, BMDCs were generated, fluorescently pre-labelled and used in *in vitro* migration assays as described before [14]. Fluorescent migrating cells were quantified using a Molecular Imager (Bio-Rad, Hercules, CA).

### *In vivo* migration of Bone Marrow derived Dendritic Cells

Mature BMDCs (mBMDC) from Jak3^+/+^-GFP^+^ and Jak3^−/−^-GFP^+^ mice were obtained and derived as described above. 1×10^6^ mBMDC were resuspended in PBS and injected into the right footpad of Jak3^+/+^ recipients. The left footpad was injected with PBS and used as control. After injection, popliteal lymph node were extracted at different time points (24, 36, 48 and 72 h), minced and analyzed by flow cytometry to identify GFP^+^ cells.

### Skin organ culture

Dorsal and ventral ear halves were obtained and rinsed twice in 70% ethanol, and once with sterile PBS. The ears halves (whole skin) were cultured in RPMI, 10% SFB. At the indicated time, pieces were carefully cut and the skin was separated into dermis and epidermis, to obtain epidermal sheets as described below. Histological sections were obtained from frozen dermis (at −80°C) and stained with an anti-mouse Langerin antibody (CD207, eBioscience, San Diego, CA) and FITC-labelled goat anti-rat IgG (H+L) antibody to identify LC in the epidermal sheets and to locate dermal cords in the dermis.

### Preparation and staining of epidermal sheets

Epidermal sheets were obtained from ears of Jak3^+/+^ and Jak3^−/−^ mice **as described** [**40**] **. Briefly, mice were sacrificed, ears were cut off at the base and the skin was separated into epidermis and dermis by using 20 mM EDTA/PBS incubation for 10 min at 65°C**. The epidermal sheets were rinsed with PBS, fixed in cold acetone and stained with anti-CD207 antibody and with a biotinylated anti I-A/I-E (2G9) antibody (Becton Dickinson), followed by streptavidin-HRP (Amersham Biotech, UK) and DAB (Zymed, Carlsbad, CA). **Dermis was snap frozen and cryostat sections were generated for further fluorescence microscopical analysis**.

### Contact Hypersensitivity (CHS) assays

2,4-dinitro-1-fluorobenzene (DNFB) was applied onto the mouse left ears at the concentration of 1% in acetone-olive oil (4∶1). Five days after sensitization, dorsal ears were challenged with 5 µl of DNFB. At 6 and 72 h after challenge ear thickness was measured using an engineer's micrometer, and compared with ear thickness prior to challenge. Ear whole skin was obtained, and epidermal sheets were prepared and stained by immunochemistry as described above. **Dermis sections were obtained as above, snap frozen and cryostat sections** were stained with haematoxylin and eosin (H&E). Morphometrical analysis was performed using the Image-Pro Plus 6.0 software (Media Cybernetics, Inc. Silver Spring, MD) and measuring five fields from each ear section and contrasting controls against challenged mice. Results were expressed as cells/mm^2^.

### Lymphocyte proliferation assays

Single cell suspensions were prepared from spleen and peripheral LN from Balb/c AnN mice as described above. B lymphocytes were depleted through panning using an anti-B220 antibody for 1 h at 37°C. 1×10^5^ T lymphocytes were labelled with CFSE (Molecular Probes, Eugene, OR) at 37°C, and seeded into 96 well plates. Mature BMDCs were obtained from Jak3^+/+^ and Jak3^−/−^ mice and co-cultured with T lymphocytes at different ratios (T lymphocytes: mBMDCs 1∶1, 1∶2, 1∶4, 1∶10 and 1∶20). After 5, 6 and 7 days of co-culture, cells were stained with PE-labelled anti-CD4 and PE-Cy5-labelled anti-CD8 (Becton Dickinson) antibodies and analyzed by Flow Cytometry as described above. Analysis was performed on CD4 or CD8 gated subpopulations. To calculate percentage of proliferation, a gate was set using unstimulated cells.

#### Statistical analysis

Unpaired two-tailed Student T-test was performed for the analysis of statistical significance; p<0.05 was considered as significant.

## Supporting Information

Figure S1 Expression of costimulatory molecules in freshly isolated DCs from Jak 3−/− mice. FACS analysis from spleen DCs from Jak3+/+ and Jak3−/− stained with anti-CD80 and anti-CD86 antibodies. Graphs represent Mean Fluorescence Intensity (MFI) of cells expressing CD80 (left) and CD86 (right), gated on CD11c+ MHCII+. Mean values ±SEM are shown (n = 11) (*p = 0.04; NS = non-significant).
(0.22 MB TIF)

Figure S2 Schematic representation of LC migration under homeostatic (A) or inflammatory (B) conditions. A) Homeostatic Conditions: LC exit slowly from epidermis in a multistep process that may take several days. 1). Molecules such as JAM-A [30] and others, such as Jak3, mediate retention of Dendritic Cells in epidermis. Under homeostatic conditions, LC leave the epidermis towards the dermis in a process independent of CCR7. 2). LC enter into the lymph vessels to initiate migration to lymph nodes. This process involves participation of molecules such as CD47 [25] and CCR7-CCL21. LC precursors present in the dermis are responsible to repopulate lymph node under steady state conditions [34]. B) Inflammatory state: LCs leave the epidermis in a coordinated process that includes several steps. 1). After sensing or capture of antigen, LCs leave the epidermis by a CCR7-independent mechanism. In this work we find that similar to this, the LCs do not need Jak3 to leave the epidermis. 2). To get entry into the lymphatic vessels and arrival to LNs, it has been demonstrated that LCs are dependent of CCR7 and other molecules as LT4 [42], MMP [43] and CXCR4 [44]. Jak3 may also be involved in this process. 3). CCR2+ GR1+ blood precursors are important to restore the LC population after inflammation. Chemokine-mediated signalling through the Jak-Stat pathway, and in particular Jak3 may also be important, as it is demonstrated in the present report
(1.40 MB TIF)

## References

[1] Banchereau J, Briere F, Caux C, Davoust J, Lebecque S (2000). Immunobiology of dendritic cells.. Annu Rev Immunol.

[2] Sallusto F, Schaerli P, Loetscher P, Schaniel C, Lenig D (1998). Rapid and coordinated switch in chemokine receptor expression during dendritic cell maturation.. Eur J Immunol.

[3] Saeki H, Moore AM, Brown MJ, Hwang ST (1999). Cutting edge: secondary lymphoid-tissue chemokine (SLC) and CC chemokine receptor 7 (CCR7) participate in the emigration pathway of mature dendritic cells from the skin to regional lymph nodes.. J Immunol.

[4] Tanabe S, Heesen M, Yoshizawa I, Berman MA, Luo Y (1997). Functional expression of the CXC-chemokine receptor-4/fusin on mouse microglial cells and astrocytes.. J Immunol.

[5] Ohl L, Mohaupt M, Czeloth N, Hintzen G, Kiafard Z (2004). CCR7 governs skin dendritic cell migration under inflammatory and steady-state conditions.. Immunity.

[6] Marsland BJ, Battig P, Bauer M, Ruedl C, Lassing U (2005). CCL19 and CCL21 induce a potent proinflammatory differentiation program in licensed dendritic cells.. Immunity.

[7] Kaiser A, Donnadieu E, Abastado JP, Trautmann A, Nardin A (2005). CC chemokine ligand 19 secreted by mature dendritic cells increases naive T cell scanning behavior and their response to rare cognate antigen.. J Immunol.

[8] Gunn MD, Kyuwa S, Tam C, Kakiuchi T, Matsuzawa A (1999). Mice lacking expression of secondary lymphoid organ chemokine have defects in lymphocyte homing and dendritic cell localization.. J Exp Med.

[9] Wong M, Uddin S, Majchrzak B, Huynh T, Proudfoot AE (2001). Rantes activates Jak2 and Jak3 to regulate engagement of multiple signaling pathways in T cells.. J Biol Chem.

[10] Mueller A, Strange PG (2004). CCL3, acting via the chemokine receptor CCR5, leads to independent activation of Janus kinase 2 (JAK2) and Gi proteins.. FEBS Lett.

[11] Stein JV, Soriano SF, M'Rini C, Nombela-Arrieta C, de Buitrago GG (2003). CCR7-mediated physiological lymphocyte homing involves activation of a tyrosine kinase pathway.. Blood.

[12] Vila-Coro AJ, Rodriguez-Frade JM, Martin De Ana A, Moreno-Ortiz MC, Martinez AC (1999). The chemokine SDF-1alpha triggers CXCR4 receptor dimerization and activates the JAK/STAT pathway.. Faseb J.

[13] Soldevila G, Licona I, Salgado A, Ramirez M, Chavez R (2004). Impaired chemokine-induced migration during T-cell development in the absence of Jak 3.. Immunology.

[14] Garcia-Zepeda EA, Licona-Limon I, Jimenez-Solomon MF, Soldevila G (2007). Janus kinase 3-deficient T lymphocytes have an intrinsic defect in CCR7-mediated homing to peripheral lymphoid organs.. Immunology.

[15] Yamaoka K, Min B, Zhou YJ, Paul WE, O'Shea J J (2005). Jak3 negatively regulates dendritic-cell cytokine production and survival.. Blood.

[16] Mellado M, Rodriguez-Frade JM, Manes S, Martinez AC (2001). Chemokine signaling and functional responses: the role of receptor dimerization and TK pathway activation.. Annu Rev Immunol.

[17] Zhang XF, Wang JF, Matczak E, Proper JA, Groopman JE (2001). Janus kinase 2 is involved in stromal cell-derived factor-1alpha-induced tyrosine phosphorylation of focal adhesion proteins and migration of hematopoietic progenitor cells.. Blood.

[18] Soldevila G, GarcÌa-Zepeda EA (2007). The role of the Jak-Stat pathway in chemokine-mediated signaling in T lymphocytes.. Signal Transduction.

[19] Kabashima K, Shiraishi N, Sugita K, Mori T, Onoue A (2007). CXCL12-CXCR4 engagement is required for migration of cutaneous dendritic cells.. Am J Pathol.

[20] Grayson MH, Ramos MS, Rohlfing MM, Kitchens R, Wang HD (2007). Controls for lung dendritic cell maturation and migration during respiratory viral infection.. J Immunol.

[21] Cappello P, Fraone T, Barberis L, Costa C, Hirsch E (2006). CC-chemokine ligand 16 induces a novel maturation program in human immature monocyte-derived dendritic cells.. J Immunol.

[22] Lutz MB, Schnare M, Menges M, Rossner S, Rollinghoff M (2002). Differential functions of IL-4 receptor types I and II for dendritic cell maturation and IL-12 production and their dependency on GM-CSF.. J Immunol.

[23] von Andrian UH, Mempel TR (2003). Homing and cellular traffic in lymph nodes.. Nat Rev Immunol.

[24] MartIn-Fontecha A, Sebastiani S, Hopken UE, Uguccioni M, Lipp M (2003). Regulation of dendritic cell migration to the draining lymph node: impact on T lymphocyte traffic and priming.. J Exp Med.

[25] Van VQ, Lesage S, Bouguermouh S, Gautier P, Rubio M (2006). Expression of the self-marker CD47 on dendritic cells governs their trafficking to secondary lymphoid organs.. EMBO J.

[26] Scandella E, Men Y, Gillessen S, Forster R, Groettrup M (2002). Prostaglandin E2 is a key factor for CCR7 surface expression and migration of monocyte-derived dendritic cells.. Blood.

[27] Scandella E, Men Y, Legler DF, Gillessen S, Prikler L (2004). CCL19/CCL21-triggered signal transduction and migration of dendritic cells requires prostaglandin E2.. Blood.

[28] Robbiani DF, Finch RA, Jager D, Muller WA, Sartorelli AC (2000). The leukotriene C(4) transporter MRP1 regulates CCL19 (MIP-3beta, ELC)-dependent mobilization of dendritic cells to lymph nodes.. Cell.

[29] Randolph GJ, Angeli V, Swartz MA (2005). Dendritic-cell trafficking to lymph nodes through lymphatic vessels.. Nat Rev Immunol.

[30] Cera MR, Del Prete A, Vecchi A, Corada M, Martin-Padura I (2004). Increased DC trafficking to lymph nodes and contact hypersensitivity in junctional adhesion molecule-A-deficient mice.. J Clin Invest.

[31] Xu H, Guan H, Zu G, Bullard D, Hanson J (2001). The role of ICAM-1 molecule in the migration of Langerhans cells in the skin and regional lymph node.. Eur J Immunol.

[32] Nishio H, Matsui K, Tsuji H, Tamura A, Suzuki K (2001). Immunolocalisation of the janus kinases (JAK)—signal transducers and activators of transcription (STAT) pathway in human epidermis.. J Anat.

[33] Mori S, Nakano H, Aritomi K, Wang CR, Gunn MD (2001). Mice lacking expression of the chemokines CCL21-ser and CCL19 (plt mice) demonstrate delayed but enhanced T cell immune responses.. J Exp Med.

[34] Merad M, Manz MG, Karsunky H, Wagers A, Peters W (2002). Langerhans cells renew in the skin throughout life under steady-state conditions.. Nat Immunol.

[35] Kissenpfennig A, Henri S, Dubois B, Laplace-Builhe C, Perrin P (2005). Dynamics and function of Langerhans cells in vivo: dermal dendritic cells colonize lymph node areas distinct from slower migrating Langerhans cells.. Immunity.

[36] Ginhoux F, Tacke F, Angeli V, Bogunovic M, Loubeau M (2006). Langerhans cells arise from monocytes in vivo.. Nat Immunol.

[37] Idoyaga J, Moreno J, Bonifaz L (2007). Tumor cells prevent mouse dendritic cell maturation induced by TLR ligands.. Cancer Immunol Immunother.

[38] Inaba K, Inaba M, Romani N, Aya H, Deguchi M (1992). Generation of large numbers of dendritic cells from mouse bone marrow cultures supplemented with granulocyte/macrophage colony-stimulating factor.. J Exp Med.

[39] Andrews DM, Andoniou CE, Granucci F, Ricciardi-Castagnoli P, Degli-Esposti MA (2001). Infection of dendritic cells by murine cytomegalovirus induces functional paralysis.. Nature Immunology.

[40] Stoitzner P, Pfaller K, StoÈssel H, Romani N (2002). A Close-Up View of Migrating Langerhans Cells in the Skin.. J Invest Dermatol.

[41] Yao Q, Doan LX, Zhang R, Bharadwaj U, Li M (2007). Thymosin-alpha1 modulates dendritic cell differentiation and functional maturation from human peripheral blood CD14+ monocytes.. Immunol Lett.

[42] Del Prete A, Shao WH, Mitola S, Santoro G, Sozzani S (2006). Regulation of Dendritic Cell Migration and Adaptive Immune Response by Leukotriene B4 Receptors: A Role for LTB4 in Up-regulation of CCR7 Expression and Function.. Blood.

[43] Ratzinger G, Stoitzner P, Ebner S, Lutz MB, Layton GT (2002). Matrix metalloproteinases 9 and 2 are necessary for the migration of Langerhans cells and dermal dendritic cells from human and murine skin.. J Immunol.

[44] Kabashima K, Sugita K, Shiraishi N, Tamamura H, Fujii N (2007). CXCR4 engagement promotes dendritic cell survival and maturation.. Biochem Biophys Res Commun.

