# Supplementary figures and images for: Jak3 Is Involved in Dendritic Cell Maturation and CCR7-Dependent Migration
Source: PLoS One. 2009 Sep 17;4(9):e7066. doi: 10.1371/journal.pone.0007066 (PMC2738966; doi:10.1371/journal.pone.0007066)

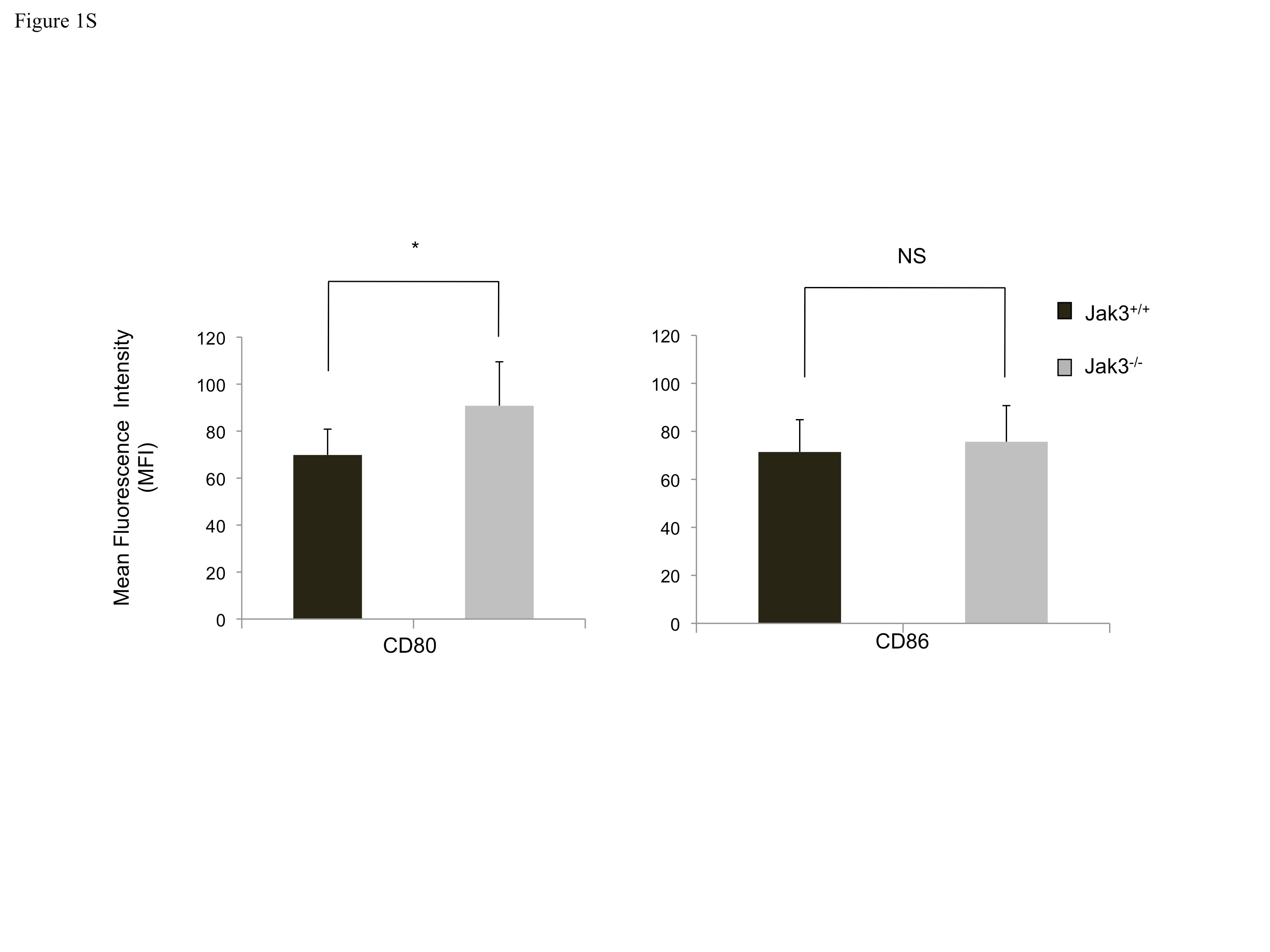

Supplement: Figure S1 — Expression of costimulatory molecules in freshly isolated DCs from Jak 3−/− mice. FACS analysis from spleen DCs from Jak3+/+ and Jak3−/− stained with anti-CD80 and anti-CD86 antibodies. Graphs represent Mean Fluorescence Intensity (MFI) of cells expressing CD80 (left) and CD86 (right), gated on CD11c+ MHCII+. Mean values ±SEM are shown (n = 11) (*p = 0.04; NS = non-significant). (0.22 MB TIF) [file pone.0007066.s001.tif]

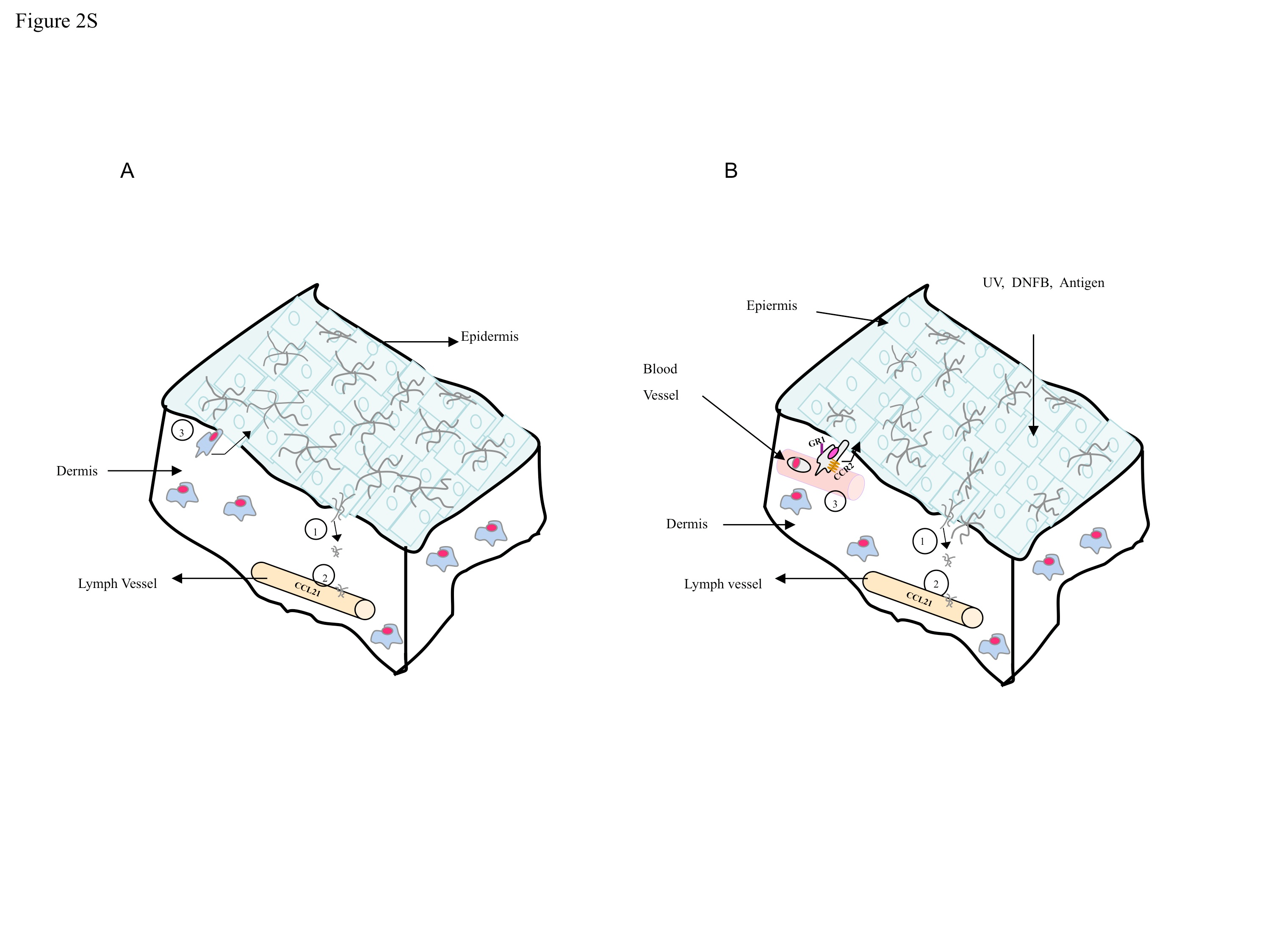

Supplement: Figure S2 — Schematic representation of LC migration under homeostatic (A) or inflammatory (B) conditions. A) Homeostatic Conditions: LC exit slowly from epidermis in a multistep process that may take several days. 1). Molecules such as JAM-A [30] and others, such as Jak3, mediate retention of Dendritic Cells in epidermis. Under homeostatic conditions, LC leave the epidermis towards the dermis in a process independent of CCR7. 2). LC enter into the lymph vessels to initiate migration to lymph nodes. This process involves participation of molecules such as CD47 [25] and CCR7-CCL21. LC precursors present in the dermis are responsible to repopulate lymph node under steady state conditions [34]. B) Inflammatory state: LCs leave the epidermis in a coordinated process that includes several steps. 1). After sensing or capture of antigen, LCs leave the epidermis by a CCR7-independent mechanism. In this work we find that similar to this, the LCs do not need Jak3 to leave the epidermis. 2). To get entry into the lymphatic vessels and arrival to LNs, it has been demonstrated that LCs are dependent of CCR7 and other molecules as LT4 [42], MMP [43] and CXCR4 [44]. Jak3 may also be involved in this process. 3). CCR2+ GR1+ blood precursors are important to restore the LC population after inflammation. Chemokine-mediated signalling through the Jak-Stat pathway, and in particular Jak3 may also be important, as it is demonstrated in the present report (1.40 MB TIF) [file pone.0007066.s002.tif]
